# Genome-Wide Association Study of Kernel Traits Using a 35K SNP Array in Bread Wheat (*Triticum aestivum* L.)

**DOI:** 10.3389/fpls.2022.905660

**Published:** 2022-06-06

**Authors:** Peng Wang, Tian Tian, Jingfu Ma, Yuan Liu, Peipei Zhang, Tao Chen, Fahimeh Shahinnia, Delong Yang

**Affiliations:** ^1^State Key Laboratory of Aridland Crop Science, Lanzhou, China; ^2^College of Life Science and Technology, Gansu Agricultural University, Lanzhou, China; ^3^College of Agronomy, Gansu Agricultural University, Lanzhou, China; ^4^Bavarian State Research Center for Agriculture, Institute for Crop Science and Plant Breeding, Freising, Germany

**Keywords:** bread wheat, kernel traits, SNP array, GWAS, candidate genes

## Abstract

Kernel size and weight are crucial components of grain yield in wheat. Deciphering their genetic basis is essential for improving yield potential in wheat breeding. In this study, five kernel traits, including kernel length (KL), kernel width (KW), kernel diameter ratio (KDR), kernel perimeter (KP), and thousand-kernel weight (TKW), were evaluated in a panel consisting of 198 wheat accessions under six environments. Wheat accessions were genotyped using the 35K SNP iSelect chip array, resulting in a set of 13,228 polymorphic SNP markers that were used for genome-wide association study (GWAS). A total of 146 significant marker-trait associations (MTAs) were identified for five kernel traits on 21 chromosomes [–log_10_(*P*) ≥ 3], which explained 5.91–15.02% of the phenotypic variation. Of these, 12 stable MTAs were identified in multiple environments, and six superior alleles showed positive effects on KL, KP, and KDR. Four potential candidate genes underlying the associated SNP markers were predicted for encoding ML protein, F-box protein, ethylene-responsive transcription factor, and 1,4-α-glucan branching enzyme. These genes were strongly expressed in grain development at different growth stages. The results will provide new insights into the genetic basis of kernel traits in wheat. The associated SNP markers and predicted candidate genes will facilitate marker-assisted selection in wheat breeding.

## Introduction

Bread wheat (*Triticum aestivum* L., 2*n* = 6 × = 42, BBAADD) is one of the most important cereals worldwide, providing calories and proteins for ∼40% of the world population (Rajaram, 2001; Golan et al., 2015). Over the past 40 years, wheat production has increased significantly, but the rate of increase in annual wheat production has tended to decline. Current increases in wheat production are likely to be insufficient to meet the food needs of the global population by 2050 (Ray et al., 2012; Ray et al., 2013). Given the increasing mismatch between acreage and world population, increasing wheat yield has become particularly crucial (Li M. et al., 2015). Thousand kernel weight (TKW), as one of the three essential components of wheat yield, has relatively higher heritability than the number of spikes per plant and the number of grains per spike. Therefore, genetic improvement of TKW is a viable approach to increase wheat yield. Kernel length (KL), kernel width (KW), kernel diameter ratio (KDR), and kernel perimeter (KP) determine kernel size and affect kernel weight and milling quality (Gegas et al., 2010; Ramya et al., 2010). Therefore, understanding the genetic basis of kernel traits is of great importance for improving wheat yield.

Most agronomic traits of wheat are complex quantitative traits controlled by multiple genes. Their genetic structure is difficult to decipher using conventional methods, so complementing them with molecular approaches is essential (Ayoub et al., 2002; Börner et al., 2002). Quantitative trait locus (QTL) mapping based on linkage analysis has been widely used to analyze the molecular genetic basis of complex quantitative traits. However, this method is only applicable to bi-parental populations and leads to low-resolution mapping of the QTLs and decreases the usefulness of detected QTLs in breeding practices. Alternatively, genome-wide association study (GWAS) provides an effective strategy to identify associations between genotypes and phenotypes. Compared with the former, GWAS can shorten breeding years because segregation population do not need to be established, broader genetic variations can be explored, and much higher accuracy in QTL detection can be achieved (Zhang P. et al., 2021). In recent years, with the development of molecular DNA markers and high-throughput genotyping technology, the single-nucleotide polymorphism (SNP) array has become a powerful tool for marker-assisted selection (MAS). GWAS using SNP chip arrays has been applied to a variety of crops such as rice (*Oryza sativa* L.) (Yang et al., 2014; Yano et al., 2016), maize (*Zea mays* L.) (Liu et al., 2016; Zhu et al., 2018), cotton (*Gossypium* spp.) (Su et al., 2018, 2020a), and other crops. GWAS is also an efficient approach for the genetic analysis of complex quantitative traits in wheat and has been reported for various traits, such as disease resistance (Riaz et al., 2017; Juliana et al., 2018; Mourad et al., 2018; Yang et al., 2019) and quality-related traits (Kristensen et al., 2018; Li et al., 2018; Liu et al., 2018; Chen et al., 2019). Pang et al. (2020) reported 90 marker-trait associations (MTAs) and identified eight putative candidate genes for TKW, KL, KW, and KDR using GWAS with 768 wheat cultivars. In addition, several studies have used the GWAS method to identify MTAs for wheat kernel traits and to determine the underlying candidate genes and function markers (Sun et al., 2017; Li et al., 2019; Alemu et al., 2020; Soumya et al., 2021). However, due to the complexity and large size of the wheat genome, the genetic basis of kernel traits is still poorly understood. In this study, we performed a GWAS for five kernel traits in six environments using the 35K SNP iSelect chip array. Our main objectives were to identify MTAs and search for useful SNP markers and potential candidate genes associated with kernel traits in wheat to be used for marker-assisted selection and genetic improvement of grain size and weight in wheat breeding.

## Materials and Methods

### Plant Materials and Field Conditions

A diverse set of 198 bread wheat accessions was used for this study. Of these, 183 varieties were from eight provinces in China, including 114 from Gansu, 33 from Shanxi, 11 from Hebei, ten from Beijing, six from Shaanxi, six from Shangdong, two from Tianjin, and one from Henan. The remaining 15 cultivars were from America (Supplementary Table 1). All cultivars were grown in six environments with different locations and years at Yuzhong farm station, Lanzhou, Gansu (35°51′N, 104°07′E; altitude 1900 m) in 2015–2016 (E1) and 2017–2018 (E3), and Tongwei farm station, Dingxi, Gansu (35°11′N, 105°19′E; altitude 1750 m) in 2015–2016 (E2), 2017–2018 (E4), 2018–2019 (E5), and 2019–2020 (E6). The two cultivation sites are characterized by a typical arid inland climate in Northwestern China, where the annual average temperature is about 7.0°C, the annual rainfall is less than 400 mm, with nearly 60% falling from July to September, but the annual evaporation capacity is more than 1,500 mm (Miao et al., 2022). A randomized complete block design was conducted with three replications, where the row length of each plot was 1 m and the row spacing was 20 cm, and 60 seeds were sown in each row. Local wheat cultivation practices were considered in field management.

### Trait Measurement and Statistical Analysis

At grain maturity, five individual plants of each cultivar were randomly selected for threshing, and incomplete seeds were removed. Kernel length (KL), kernel width (KW), kernel diameter ratio (KDR), kernel perimeter (KP), and thousand kernel weight (TKW) were measured by image analysis using the SC-G software (Hangzhou Wanshen Detection Technology Co., Ltd., Hangzhou, China). All trait measurements were repeated three times.

The best linear unbiased prediction (BLUP) of kernel traits for wheat was calculated by using the R package “lme4.” The broad-sense heritability (*h*^2^_B_) was calculated by the following equation (Sukumaran et al., 2015): *h*^2^_B_ = σ^2^_G_/(σ^2^_G_ + σ^2^_G × E_/l + σ^2^_e_/rl), where σ^2^_G_ is the genetic variance, σ^2^_G × E_ is the genotype-environment variance, σ^2^_e_ is the residual variance, l is the number of environments, and r is the number of replications. Descriptive statistics, ANOVA, and Pearson’s correlation coefficient were calculated using IBM SPSS Statistics V.25.

### Single-Nucleotide Polymorphism Genotyping and Population Structure Analysis

A panel of 198 wheat accessions was genotyped by the 35K SNP iSelect chip array (Beijing Zhongyujin Marking Co., Ltd., Beijing, China), which contained 35,143 SNP markers (Allen et al., 2017). The markers without clear physical position information on the chromosomes were removed. The genotyping data were filtered by removing markers with missing values > 20% and minor allele frequency (MAF) < 5%. As a result, 13,228 polymorphic SNP markers were used for subsequent analysis.

The ADMIXTURE V1.3 software was applied to infer population structure based on the filtered polymorphic SNP markers. The genotypes were clustered by assuming the number of groups with *K* value set as 2–10. The optimal number of clusters was determined according to the cross-validation error (CV error) rate, and the *K*-value with the minimum CV error rate corresponds to the optimal number of subpopulations (Alexander et al., 2009). A neighbor-joining (NJ) phylogenetic tree of genotypes was constructed by the PowerMarker V3.25 software (Liu and Muse, 2005).

### Genome-Wide Association Analysis

The GWAS analysis was conducted by the TASSEL V5.0 software using the mixed linear model (Q + K, MLM), where population structure (Q) and kinship (K) matrices were applied to avoid spurious associations caused by population structure and control false positives simultaneously. To declare the significant MTAs, *P*-value after Bonferroni correction was considered with high stringency. Therefore, *P* ≤ 0.001 [–log_10_(*P*) ≥ 3] was regarded as the threshold using a liberal approach to reduce ignoring any significantly associated SNP marker for the kernel traits (Kumar et al., 2020). Manhattan plots and Quantile-Quantile (Q-Q) plots were drawn using the R package ‘‘CMplot.’’^1^

### Identification of Candidate Genes

After the significant MTAs were identified in multiple environments, the flanking sequence spanning 1 kb upstream and downstream of the significant SNP position was used to query against the Chinese Spring wheat reference genome IWGSC RefSeq v1.1.^2^ Subsequently, JBrowse was used to examine candidate genes that overlapped with the flanking region of the SNPs (Kumar et al., 2020). Functional annotations of candidate genes were extracted from WheatGmap^3^ (Zhang L. et al., 2021). To investigate the function of candidate genes, expression analysis was performed based on the publicly available expression data of a drought-tolerant cultivar Jinmai 47, obtained from roots, stems, leaves, developing spikes, spikes at the anthesis stage, and developing grains at 5, 10, and 20 days after anthesis (DAA). Total RNA was extracted using the E.Z.N.A.^®^ Plant RNA Kit (Omega Bio-Tek, Norcross, GA, United States). Three biological replications were used for quantitative real-time PCR (qRT-PCR) analysis in each treatment. *TaActin* was used as an endogenous reference, and the 2^–ΔΔC(t)^ method was used to determine relative gene expression levels. Primer sequences used for qRT-PCR are listed in Supplementary Table 2.

## Results

### Phenotypic Evaluation

**FIGURE 1:**
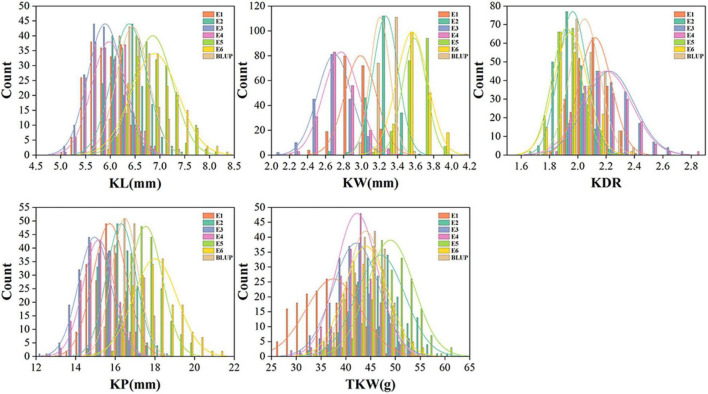
Distribution of the kernel traits in 198 wheat association panel. KL, kernel length; KW, kernel width; KDR, kernel diameter ratio; KP, kernel perimeter; TKW, thousand kernel weight.

Phenotypic performance of kernel traits showed wide variation and normal distribution in different environments (Figure 1). The coefficients of variation for each trait in different environments ranged from 3.44 to 14.79%, indicating broad phenotypic variation among genotypes. The broad-sense heritabilities of KL, KW, KDR, KP, and TKW were moderate to high, ranging from 0.60 to 0.85. In comparison, KW had the lowest *h*^2^_B_ value (0.60), whereas KL had the highest *h*^2^_B_ value (0.85) (Table 1).

**TABLE 1:** Descriptive statistics and heritability (*h*^2^_B_) of five kernel traits.

Trait	Environment	Min	Max	Mean	SD	CV^a^ (%)	*F*-test^b^	h^2^_B_^c^
KL (mm)	E1	5.22	7.36	6.28	0.38	5.99	34.77**	
	E2	5.47	7.48	6.38	0.36	5.63	61.53**	
	E3	5.02	7.00	5.90	0.37	6.23	19.95**	
	E4	5.14	7.11	5.97	0.36	6.06	35.39**	
	E5	6.06	8.06	6.86	0.40	5.84	219.60**	
	E6	5.92	8.36	6.89	0.48	6.95	340.49**	
	BLUP	5.80	7.33	6.45	0.33	5.05	231.37**	0.85
KW (mm)	E1	2.29	3.47	2.99	0.18	6.18	20.03**	
	E2	2.64	3.63	3.27	0.15	4.44	22.37**	
	E3	2.01	3.29	2.70	0.21	7.63	10.53**	
	E4	2.23	3.42	2.77	0.21	7.40	21.13**	
	E5	3.14	3.87	3.59	0.13	3.73	72.20**	
	E6	3.00	4.04	3.56	0.18	4.92	134.71**	
	BLUP	2.84	3.44	3.21	0.11	3.44	58.96**	0.60
KDR	E1	1.85	2.49	2.12	0.12	5.54	59.42**	
	E2	1.72	2.35	1.96	0.10	5.19	75.06**	
	E3	1.87	2.94	2.23	0.18	8.04	32.97**	
	E4	1.83	2.92	2.20	0.19	8.46	25.41**	
	E5	1.69	2.23	1.92	0.11	5.47	208.48**	
	E6	1.69	2.35	1.95	0.12	6.04	220.81**	
	BLUP	1.84	2.35	2.05	0.11	5.22	153.76**	0.80
KP (mm)	E1	12.32	17.95	15.71	0.87	5.54	35.93**	
	E2	14.76	18.56	16.30	0.77	4.70	54.31**	
	E3	11.78	17.30	14.96	0.90	5.99	10.93**	
	E4	12.94	17.39	15.16	0.83	5.47	19.17**	
	E5	15.75	19.97	17.54	0.84	4.78	153.79**	
	E6	15.53	21.27	18.01	1.11	6.17	197.88**	
	BLUP	14.88	18.26	16.48	0.68	4.12	133.44**	0.79
TKW (g)	E1	26.80	51.85	37.49	5.55	14.79	258.23**	
	E2	32.95	62.19	47.01	4.97	10.57	144.18**	
	E3	29.87	56.12	42.09	4.70	11.17	170.71**	
	E4	28.72	53.51	42.24	4.24	10.03	103.50**	
	E5	34.55	61.72	49.02	4.78	9.76	128.47**	
	E6	32.40	55.71	44.05	4.38	9.94	168.24**	
	BLUP	32.88	54.14	44.00	3.70	8.40	515.26**	0.80

*^a^CV, coefficient of variation.*

^b^**, P ≤ 0.01.

*^c^h^2^_B_, the broad sense heritability.*

**FIGURE 2:**
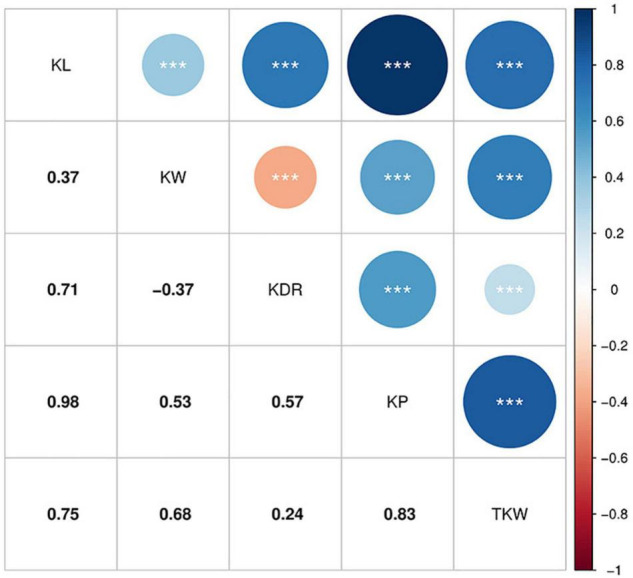
Pearson’s correlation coefficients among BLUP for the kernel traits. ***Significant at *P* < 0.001.

Pearson’s correlation coefficients based on BLUP for the kernel traits are shown in Figure 2. All traits were significantly correlated with each other (*P* < 0.001). KL, KW, KDR, and KP showed positive correlations with TKW (*r* = 0.24–0.83). KW showed a negative correlation with KDR (*r* = –0.37), whereas the other traits were positively correlated with each other. The highest correlation was between KL and KP (*r* = 0.98).

### Marker Distribution and Population Structure

A total of 35,143 SNPs were genotyped for 198 wheat accessions. After filtering, 13,228 SNPs were polymorphic and were used for subsequent analysis (Supplementary Figure 1). Of these, 4,704, 5,408, and 3,116 SNPs were observed in the A, B, and D sub-genomes, respectively. On average, 1,890 SNP markers were distributed per homeologous group. The smallest number of SNPs was found in the homeologous group IV (1,070 SNPs), and the largest number of SNPs was found in the homeologous group II (2,519 SNPs). The distribution of SNP markers varied greatly across 21 chromosomes, with the fewest SNPs on chromosome 4D (180 SNPs) and the most on chromosome 2B (980 SNPs).

**FIGURE 3:**
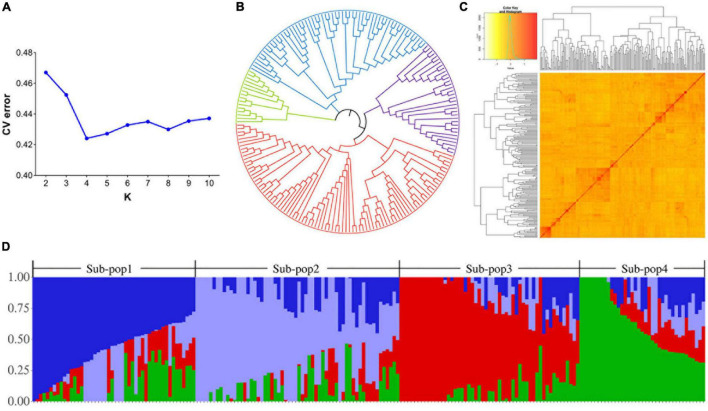
Population structure and kinship analyses of the 198 wheat accessions. **(A)** Graph of the cross-validation of errors. The numbers of clusters (K) were set from 2 to 10. **(B)** A neighbor-joining (NJ) phylogenetic tree of the 198 wheat accessions. **(C)** Clustering heat map created using kinship matrix. **(D)** Four subpopulations for 198 wheat accessions. The X-axis represents the number of accessions, and the Y-axis shows the membership probabilities of the subpopulations.

The inferring population structure showed a minimum CV error rate value at *K* = 4, indicating that the association population was structured into four subpopulations. Each of them contained 48, 60, 44, and 46 accessions, respectively (Figures 3A,D and Supplementary Table 1). Genetic clustering with the kinship matrix and the NJ phylogenetic tree also showed that the association population could be clustered into four subpopulations (Figures 3B,C). Thus, it was suitable to divide the association population into four subpopulations.

### Marker-Trait Associations

**FIGURE 4:**
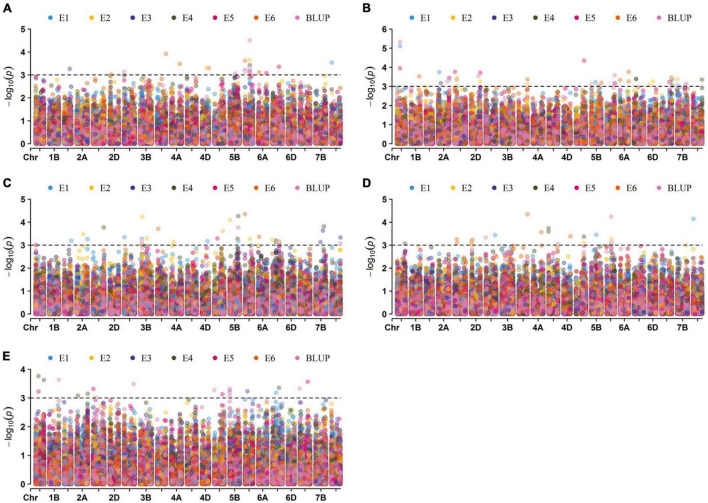
Manhattan plots for the kernel traits. The association of 13228 SNPs located on 21 chromosomes in six environments and BLUP for KL, KW, KDR, KP, and TKW were shown in panels **(A–E)**, respectively. Dotted lines indicate the threshold value at –log_10_(*P*) = 3.

A total of 146 significant MTAs (*P* ≤ 0.001) for the kernel traits were identified on all 21 chromosomes, explaining phenotypic variation (*R*^2^) ranging from 5.91 to 15.02% (Supplementary Table 3 and Figure 4). The overall rate of false-positive association results was properly controlled, as shown by quantile-quantile plots (Supplementary Figure 2). Of these, 22, 43, 36, 23, and 22 MTAs were identified for KL, KW, KDR, KP, and TKW, respectively, in all environments (Supplementary Figure 3).

In total, twelve MTAs were repeatedly observed in multiple environments, with *R*^2^ ranging from 5.91 to 15.02%. Therefore, these MTAs were considered stable loci. Of these, seven MTAs were repeatedly observed in two environments, four MTAs were repeatedly observed in three environments, and one MTA was repeatedly observed in all environments except E1 and E6. In addition, three SNP markers involved in six MTAs were associated with multiple kernel traits. Two markers, *AX-94400331* and *AX-94509671*, were both significantly associated with KL and KP, and *AX-94393836* was significantly associated with KW and TKW (Table 2).

**TABLE 2:** Significant marker-trait associations for kernel traits under multiple environments.

Trait	SNP marker	Allele^a^	Chr^b^	Postion (bp)	Environment	*P*-value	*R*^2c^ (%)
KL	*AX-95248961*	A/T	5D	6450092	E5	6.41E-04	7.91
					E6	2.40E-04	8.91
					BLUP	6.88E-04	7.80
	*AX-94400331*	C/T	5D	330891375	E4	3.77E-04	8.76
					BLUP	4.81E-04	8.44
	*AX-94509671*	A/C	5D	331402475	E2	2.25E-04	8.99
					E3	6.67E-04	7.69
					E4	4.57E-04	8.20
					E5	7.87E-04	7.57
					BLUP	3.08E-05	11.23
KW	*AX-94393836*	C/T	1A	208219190	E1	7.78E-06	14.68
					E5	1.13E-04	10.62
					BLUP	4.59E-06	15.02
	*AX-94438072*	C/T	5D	561626204	E3	7.14E-04	6.14
					E5	6.86E-04	6.36
					BLUP	2.63E-04	7.35
KDR	*AX-94409249*	A/G	3B	126000659	E1	5.21E-04	8.13
					E6	8.73E-04	7.81
					BLUP	6.46E-04	8.19
	*AX-95629937*	A/G	5B	604022658	E4	5.44E-05	11.90
					BLUP	1.72E-04	10.38
	*AX-94711022*	A/G	6B	252383444	E3	6.42E-04	6.32
					E4	7.36E-04	6.06
	*AX-94578940*	A/C	7D	627323708	E3	4.63E-04	6.58
					BLUP	8.40E-04	5.91
KP	*AX-94400331*	C/T	5D	330891375	E4	9.09E-04	7.75
					BLUP	7.48E-04	7.89
	*AX-94509671*	A/C	5D	331402475	E2	5.55E-04	7.97
					BLUP	5.72E-05	10.49
TKW	*AX-94393836*	C/T	1A	208219190	E4	1.73E-04	10.66
					E5	5.95E-04	8.68

*^a^Superior alleles are underlined.*

*^b^Chr, chromosome.*

*^c^Phenotypic variance explained by the MTAs.*

### Allelic Effects of Associated Markers on Kernel Traits

**FIGURE 5:**
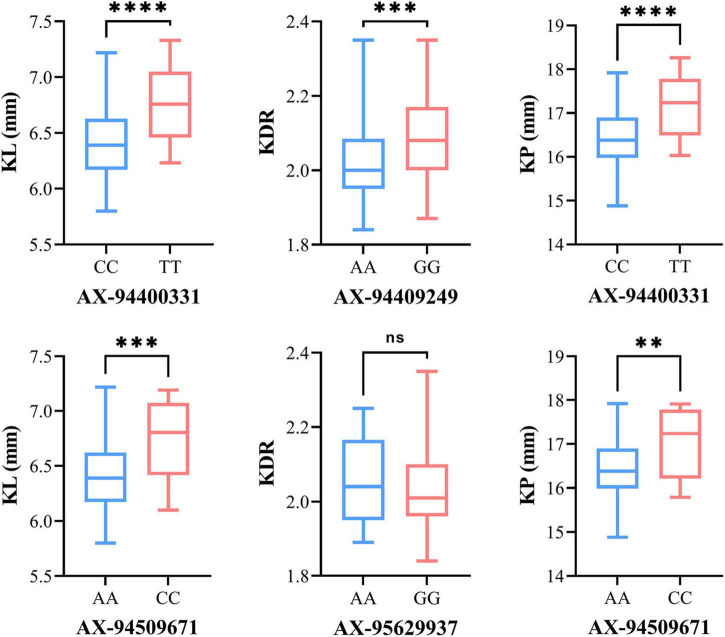
Box plot for phenotypic values of two allelic groups belonging to the associated markers identified under BLUP environments. The significant difference between the phenotype of two allelic groups was analyzed by *T*-test (^**^P ≤ 0.01; ^***^P ≤ 0.001; ^*⁣*⁣**^P ≤ 0.0001). The X-axis shows the two alleles for each SNP marker, and the Y-axis shows the phenotypic values of kernel traits.

The allelic effect was estimated from the MTAs repeatedly identified in multiple environments. Significant differences between SNP alleles were found for six MTAs except *AX-95629937* (Figure 5). The alleles of *AX-94400331* and *AX-94509671* showed positive additive effects, increasing both KL and KP. For *AX-94400331*, the allelic effect on mean KL between the superior allele TT and the inferior allele CC was 0.33 mm, and the corresponding effect on mean KP was 0.70 mm. For *AX-94509671*, the allelic effect on mean KL between the superior allele CC and the inferior allele AA was 0.32 mm, and the corresponding effect on mean KP was 0.64 mm. In addition, the mean KDR of the superior alleles (GG and AA) was slightly higher than that of the inferior alleles (AA and GG) for both markers *AX-94409249* and *AX-95629937*.

**FIGURE 6:**
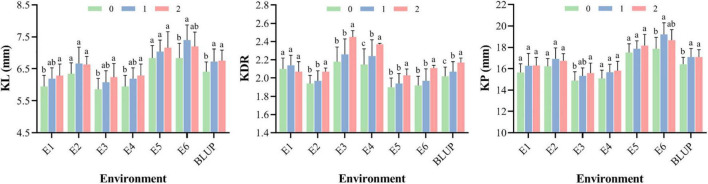
The mean of KL, KDR, and KP for wheat accessions with 0–2 superior alleles in different environments. The different letters are significantly different at *P* < 0.05 according to Duncan’s multiple range test.

The average values for phenotypes of wheat accessions carrying different numbers of superior alleles (0–2) are shown in Figure 6. Under different environments, the average KL with two superior alleles was 6.65 mm (6.24–7.20 mm). In contrast, the average KL with one superior allele and neither superior allele was 6.61 mm (6.08–7.40 mm) and 6.32 mm (5.86–6.84 mm), respectively. For KDR, the mean score with two superior alleles was 2.18 (2.03–2.45), and the mean scores with one superior allele and neither superior allele were 2.09 (1.94–2.26) and 2.03 (1.90–2.18), respectively. In addition, the average KP with different superior alleles showed the same result as KL, indicating that the phenotypic values for these kernel traits are higher when they contain more superior alleles.

### Candidate Genes for Kernel Traits

**FIGURE 7:**
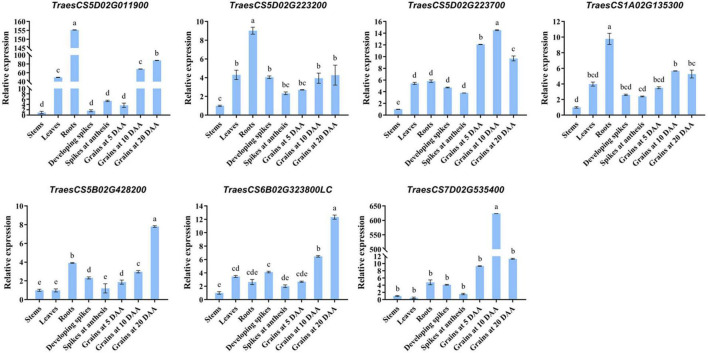
Relative expression level of different tissues obtained from qRT-PCR analysis. Roots, stems, and leaves were collected at the three-leaf stage. Spikes were collected at developing (length: 40–60 mm) and anthesis. Grains were collected at 5, 10, and 20 days after anthesis (DAA). Data are the mean ± standard deviation of three biological replicates. The different letters are significantly different at *P* < 0.05 according to Duncan’s multiple range test.

By using the flanking sequence alignment of significant SNP markers from 12 stable MTAs, nine candidate genes for the kernel traits were identified, controlling diverse functional groups of proteins (Table 3). Functional annotations showed that four KDR-associated candidate genes, namely, *TraesCS3B02G139500* on chromosome 3B, *TraesCS5B02G428200* on chromosome 5B, *TraesCS6B02G323800LC* on chromosome 6B, and *TraesCS7D02G535400* on chromosome 7D. Those genes encoded a rotundifolia-like protein, an MD-2-related lipid recognition domain-containing protein, an F-box protein, and a 1,4-α-glucan branching enzyme, respectively. On chromosome 5D, two candidate genes, namely, *TraesCS5D02G011900* and *TraesCS5D02G657200LC*, correlated with KL and KW, and they encoded hydroxysteroid dehydrogenase and chromosomal replication initiator protein DnaA, respectively. Of the other three candidate genes, which were involved in two different traits, the gene *TraesCS5D02G223200* on chromosome 5D for KL and KP encoded serine protease HtrA-like and the gene *TraesCS5D02G223700* on chromosome 5D for KL and KP encoded ethylene-responsive transcription factor. *TraesCS1A02G135300* on chromosome 1A for KW and TKW encoded a protein of the superfamily of nucleoside triphosphate hydrolases with P-loop. The expression levels of the candidate genes were examined in various tissues, including roots, stems, leaves, developing spikes, spikes at anthesis, and developing grains at 5, 10, and 20 DAA. Among the nine candidate genes identified, the CDS sequences of *TraesCS5D02G657200LC* and *TraesCS5B02G428200* were short, and it was difficult to design suitable specific primers. Therefore, we only presented the qRT-PCR results for the other seven candidate genes (Figure 7). The relative expression of each gene was different at different growth stages. Among them, both *TraesCS5D02G223700* and *TraesCS7D02G535400* were strongly expressed in the developing grains at different growth stages, with the highest expression level shown at 10 DAA. Both *TraesCS5B02G428200* and *TraesCS6B02G323800LC* were strongly expressed in developing grains at 20 DAA. This suggests that the above four genes may play a key role in regulating grain development.

**TABLE 3:** Candidate genes underlying the significant markers associated with kernel traits.

Trait	SNP marker	Chr	Postion (bp)	Gene postion (bp)	Gene ID	Functional annotation
KL	*AX-95248961*	5D	6450092	6444885–6450213	*TraesCS5D02G011900*	Hydroxysteroid dehydrogenase, putative
	*AX-94400331*	5D	330891375	330889652–330899414	*TraesCS5D02G223200*	Serine protease HtrA-like
	*AX-94509671*	5D	331402475	331402389–331406349	*TraesCS5D02G223700*	Ethylene-responsive transcription factor
KW	*AX-94393836*	1A	208219190	208208304–208219356	*TraesCS1A02G135300*	P-loop containing nucleoside triphosphate hydrolases superfamily protein
	*AX-94438072*	5D	561626204	561625578–561630640	*TraesCS5D02G657200LC*	Chromosomal replication initiator protein DnaA
KDR	*AX-94409249*	3B	126000659	125995818–125996030	*TraesCS3B02G139500*	Rotundifolia-like protein
	*AX-95629937*	5B	604022658	604021148–604023499	*TraesCS5B02G428200*	MD-2-related lipid recognition domain-containing protein/ML domain-containing protein
	*AX-94711022*	6B	252383444	252385913–252388254	*TraesCS6B02G323800LC*	F-box protein (DUF295)
	*AX-94578940*	7D	627323708	627322122–627328145	*TraesCS7D02G535400*	1,4-α-glucan branching enzyme
KP	*AX-94400331*	5D	330891375	330889652–330899414	*TraesCS5D02G223200*	Serine protease HtrA-like
	*AX-94509671*	5D	331402475	331402389–331406349	*TraesCS5D02G223700*	Ethylene-responsive transcription factor
TKW	*AX-94393836*	1A	208219190	208208304–208219356	*TraesCS1A02G135300*	P-loop containing nucleoside triphosphate hydrolases superfamily protein

## Discussion

### Phenotypic Variation and Population Structure

We used image analysis technology for the phenotyping of kernel traits, which drastically reduces artificial errors and ensures the accuracy of measurement results. The phenotypic distribution of the kernel traits showed normal distribution of the traits, indicating that these traits are quantitative traits and suitable for GWAS analysis. Large phenotypic variation was observed in all target traits, of which the largest variation was observed in TKW with coefficients of variation ranging from 8.40 to 14.79% in different environments (Figure 1). In addition, TKW showed a higher *h*^2^_B_ of 0.80 (Table 1), which is consistent with previous studies (Li Q. et al., 2015; Su et al., 2018). The *h*^2^_B_ of other kernel traits was moderate to high, ranging from 0.60 to 0.85. This indicated that the traits were largely controlled by genetic factors. Correlation analysis indicated that KL, KW, KDR, and KP contributed to TKW (Figure 2), suggesting that kernel size traits should be targeted for improving kernel weight and yield in wheat.

Since the rich germplasm resources contain substantial genetic variations and superior alleles (Walsh, 1981), we collected a diverse set of bread wheat accessions from eight different ecological regions in China and America that provides the basis for identifying MTAs. To reduce spurious associations between markers and traits, it was necessary to evaluate the genetic structure of the population before performing GWAS. The presence of four subpopulations in this associated population was confirmed by population structure and diversity analyses (Figure 3).

### Stable Marker-Trait Associations

To ensure the accuracy of the association results, we used the MLM model with population structure and kinship. This approach has been widely used in previous studies and was able to efficiently control false positives (Ain et al., 2015; Zuo et al., 2019; Alemu et al., 2020). As a result, a total of 146 significant MTAs were identified for the five kernel traits (Supplementary Table 2). Of these, 12 MTAs were repeatedly detected in multiple environments (Table 2), which were considered stable MTAs. Accordingly, nine SNP markers were involved in these MTAs. Three SNP markers of them were simultaneously correlated with multiple traits. For example, two SNP markers, namely, *AX-94400331* and *AX-94509671* were significantly associated with KL and KP, and another marker, namely, *AX-94393836* was significantly associated with KW and TKW.

Some studies have performed QTL mapping for wheat kernel traits using different molecular markers (Ramya et al., 2010; Okamoto et al., 2013; Cui et al., 2014). In this study, 12 stable MTAs were identified and compared with earlier studies while taking into account the correlations among traits, of which five MTAs were co-localized with QTLs for kernel traits previously reported. The SNP marker *AX-94393836* associated with KW and TKW on chromosome 1A was mapped within the confidence interval (*wmc24*∼*wmc278*) of a TKW QTL (*QTkw.ncl-1A.1*) reported by Ramya et al. (2010) and co-localized with a QTL (*QTkw-1A.1*) for TKW reported by Cui et al. (2014). Both *AX-95629937* and *AX-94578940* that were associated with KDR co-localized with *QTkw-5B.1* on chromosome 5B for KL and *QTkw-7D.2* on chromosome 7D for TKW, respectively, as reported by Cui et al. (2014). In addition, the KL locus *AX-95248961* on chromosome 5D was near to a QTL (*QTkw-7D.2*) for hundred grain weight reported in Okamoto et al. (2013). Notably, the remaining seven MTAs were not reported previously and are likely novel loci for kernel traits.

### Discovery of Superior Alleles

In this study, we identified 12 stable MTAs in multiple environments based on the GWAS results. The superior alleles identified from these MTAs were of greater importance. Therefore, we compared the average BLUP value of phenotype regulated by superior and inferior alleles based on six MTAs, and all superior alleles showed positive effects on KL, KP, and KDR. In particular, the superior allele TT for *AX-94400331* and CC for *AX-94509671* simultaneously showed highly positive effects on KL and KP (Figure 6).

Exploring superior alleles is very valuable for wheat breeding programs. Although the contribution of a single marker in influencing phenotypic variation might be small, the combination of superior alleles from different markers can have much larger effects in a single variety (Li et al., 2020). In our study, phenotype scores for KL, KDR, and KP were found to be positively correlated with the number of superior alleles, suggesting that pyramiding superior alleles improved wheat kernel traits performance. With the development of sequencing technology, allelic pyramiding becomes more feasible and powerful.

### Potential Candidate Genes for the Kernel Traits

In wheat, several genes have been identified that are related to kernel size and weight. For example, *TaCYP78A3*, which encodes cytochrome P450 CYP78A3, has a significant effect on kernel size by affecting seed coat cell number, and its silencing can cause an 11% decrease in wheat kernel size (Ma et al., 2015). In addition, *TaGW2* encodes E3 RING ligase, which affects thousand kernel weight by influencing kernel length and width (Simmonds et al., 2016; Zhang et al., 2018). In this study, a total of nine candidate genes were functionally annotated based on the Chinese Spring wheat reference genome (IWGSC RefSeq v1.1) (Table 3). Using qRT-PCR analysis, four promising genes were strongly expressed in developing grain at different growth stages (Figure 7), which were considered as potential candidate genes to control kernel traits.

We predicted four potential candidate genes for kernel traits. The marker *AX-95629937* was associated with KDR in E4 and BLUP, with a potential candidate gene *TraesCS5B02G428200* on chromosome 5B encoding the MD-2-related lipid recognition/ML domain-containing protein. The MD-2-related lipid recognition domain is defined as an ML protein (Inohara and Nuñez, 2002). Hakenjos et al. (2013) identified ML3 as a NEDD8- and ubiquitin-modified protein in Arabidopsis (*Arabidopsis thaliana*), which was also conjugated to ubiquitin pathway with a critical role in controlling seed size in plants (Li and Li, 2014).

The gene *TraesCS6B02G323800LC* on chromosome 6B underlying the marker *AX-94711022* was associated with KDR in E3 and E4 environments. The gene encoded the F-box protein, which is involved in diverse hormone signal transduction and cellular processes (Kuroda et al., 2002). Li et al. (2011) found that *LARGER PANICLE* (*LP*), which encodes an F-box protein and is involved in the regulation of cytokinin levels, can improve rice yield by regulating panicle structure.

The marker *AX-94509671* was associated with KL in all environments except E1 and E6 and with KP in E2 and BLUP. A potential candidate gene *TraesCS5D02G223700* encoding ethylene-responsive transcription factor (ERF) was found near the physical map position of *AX-94509671* on chromosome 5D. ERF is a plant-specific transcription factor involved in plant growth and development processes (Jin and Liu, 2008). Xu et al. (2016) reported that *OsERF* interacts with a transcription factor (*OsNF-YB1*) specifically expressed in the aleurone layer of the endosperm. This process affected kernel filling and endosperm development, regulating rice kernel size and weight. Li et al. (2017) found that the transcription factor *ZmEREB94* regulates several starch-synthetic genes, further affecting endosperm development.

The marker *AX-94578940* associated with KDR in the E3 environment and BLUP corresponded to a candidate gene *TraesCS7D02G535400* (7D) encoding the 1,4-α-glucan branching enzyme (GBE). GBE is the key enzyme that catalyzes the formation of α-1,6-linked branch in starch and belongs to the glycoside hydrolase 13 (GH13) family (Xia et al., 2021). In tapioca and maize, GBE treatment led to molecular restructuring of starch, resulting in a change in the amylose and amylopectin content of starch. This suggests that GBE is important for starch modification (Li et al., 2016a,b; Ren et al., 2018). We speculated that the above four genes might be very important in influencing kernel size and weight in wheat.

## Conclusion

In this study, GWAS for kernel traits was performed on a diverse panel of 198 bread wheat accessions, genotyped with the 35K SNP iSelect chip array. As a result, 146 significant MTAs were identified for five kernel traits. Of these, 12 stable significant MTAs were identified in different environments. Six superior alleles of nine major SNP markers showed positive effects on KL, KP, and KDR. Four potential candidate genes were predicted to be highly expressed in the developing grain at different growth stages. The results provide not only new insights into the genetic basis of kernel traits in wheat but also diagnostic makers that can be used for marker-assisted selection in wheat breeding.

## Data Availability Statement

The original contributions presented in this study are included in the article/Supplementary Material, further inquiries can be directed to the corresponding author.

## Author Contributions

PW and DY conceived the study. PW, TT, and JM performed the phenotypic data measurement and data analysis. YL, PZ, TC, and FS provided scientific feedback and revised the content. PW wrote the first draft of the manuscript. DY and FS revised and edited the manuscript. All authors have read and agreed to the published version of the manuscript.

## Conflict of Interest

The authors declare that the research was conducted in the absence of any commercial or financial relationships that could be construed as a potential conflict of interest.

## Publisher’s Note

All claims expressed in this article are solely those of the authors and do not necessarily represent those of their affiliated organizations, or those of the publisher, the editors and the reviewers. Any product that may be evaluated in this article, or claim that may be made by its manufacturer, is not guaranteed or endorsed by the publisher.

## Footnotes

1
https://github.com/YinLiLin/R-CMplot

2
http://www.wheatgenome.org/

3
https://www.wheatgmap.org/
