# Supplementary material for: Genome-Wide Association Study of Kernel Traits Using a 35K SNP Array in Bread Wheat (Triticum aestivum L.)
Source: Front Plant Sci. 2022 Jun 6;13:905660. doi: 10.3389/fpls.2022.905660 (PMC9207461; doi:10.3389/fpls.2022.905660)
Supplement: Supplementary file 1 [file Data_Sheet_1.docx]

Supplementary Material

# Supplementary Figures and Tables

## Supplementary Figures


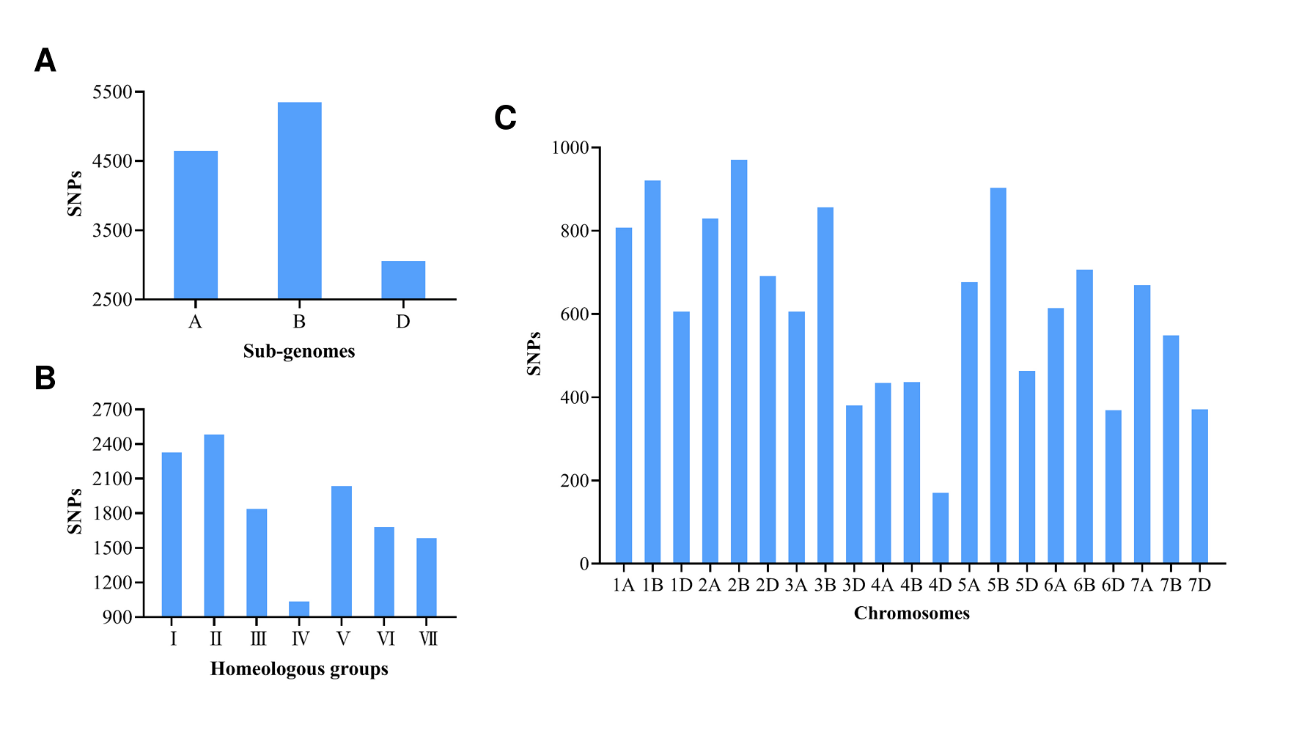


**Supplementary Figure 1.** Number of SNP markers on different sub-genomes **(A)**, homeologous groups **(B)** and chromosomes **(C)** based on 13,228 polymorphic SNPs after filtering.


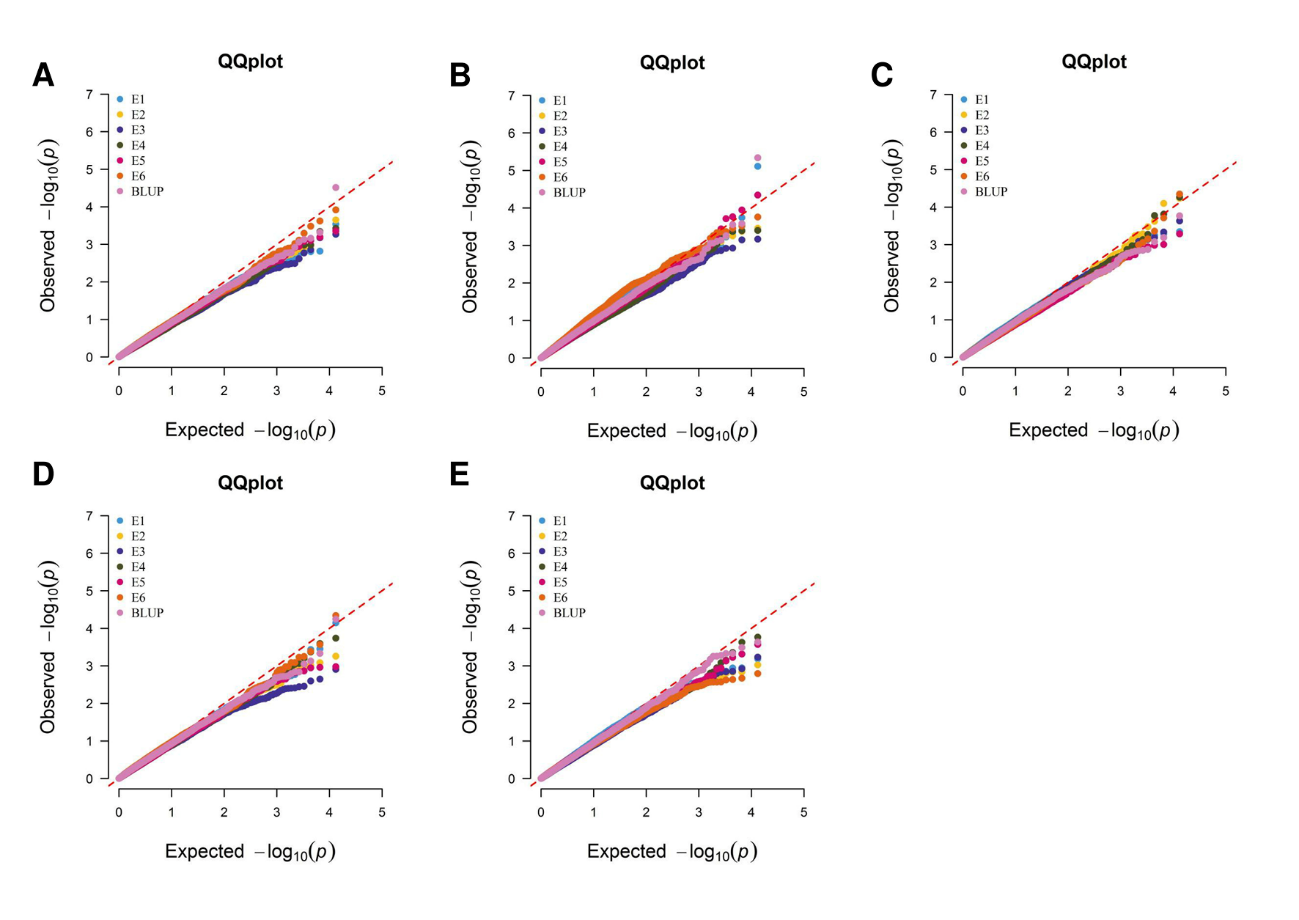


**Supplementary Figure 2.** Multi-track Q-Q plots for the kernel traits. The results of KL, KW, KDR, KP and TKW were showed in **(A)**, **(B)**, **(C)**, **(D)** and **(E)**, respectively.

**
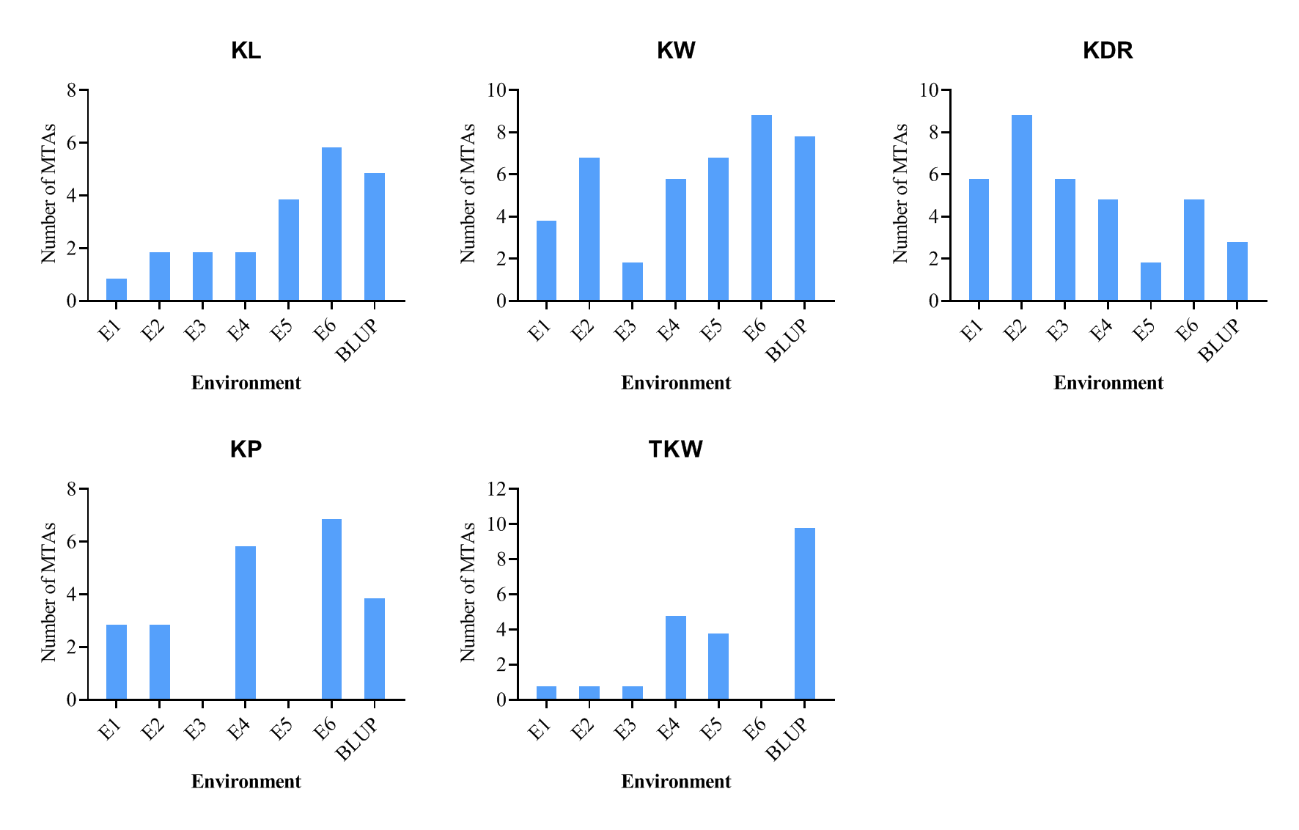
**

**Supplementary Figure 3.** Number of significant marker-trait associations for the kernel traits in different environments. The threshold value was -log10(*P*) = 3.

## Supplementary Tables

**Supplementary Table 1.** Information of the 198 wheat accessions used in this study.

| **No.** | **Name** | **Origin** | **Sub-population** |
| --- | --- | --- | --- |
| 1 | 21-30 | Gansu | Sub-pop2 |
| 2 | 22-23 | Gansu | Sub-pop1 |
| 3 | Jingdong 22 | Gansu | Sub-pop1 |
| 4 | Longyu 218 | Gansu | Sub-pop2 |
| 5 | Zhongmai 175 | Beijing | Sub-pop4 |
| 6 | Changwu 521 | Shaanxi | Sub-pop1 |
| 7 | Zhongmai 553 | Beijing | Sub-pop2 |
| 8 | Zhongyou 9507 | Beijing | Sub-pop1 |
| 9 | Jingdong 17 | Beijing | Sub-pop2 |
| 10 | 70410 | Gansu | Sub-pop1 |
| 11 | Lantian 8 | Gansu | Sub-pop1 |
| 12 | Lantian 9 | Gansu | Sub-pop1 |
| 13 | Longyuan 036 | Gansu | Sub-pop1 |
| 14 | 1389-2 | Gansu | Sub-pop1 |
| 15 | Longzhong 1 | Gansu | Sub-pop2 |
| 16 | C78-3-6-1-3 | Gansu | Sub-pop1 |
| 17 | Longjian 108 | Gansu | Sub-pop2 |
| 18 | C49-1-1-1-2 | Gansu | Sub-pop1 |
| 19 | 01-368 | Gansu | Sub-pop1 |
| 20 | C53-3-2-1-1 | Gansu | Sub-pop1 |
| 21 | 0025-17-1 | Gansu | Sub-pop1 |
| 22 | 99384-2-1 | Gansu | Sub-pop1 |
| 23 | H-4-2-2-1 | Gansu | Sub-pop1 |
| 24 | B160-2-2-1-1 | Gansu | Sub-pop1 |
| 25 | C42-2-1-1 | Gansu | Sub-pop2 |
| 26 | E71-2-6 | Gansu | Sub-pop2 |
| 27 | C47-1-1-2 | Gansu | Sub-pop1 |
| 28 | B17-2-3-1-1-2 | Gansu | Sub-pop2 |
| 29 | C42-2-3-2 | Gansu | Sub-pop4 |
| 30 | C55-8-1-3-1 | Gansu | Sub-pop2 |
| 31 | Longjian 110 | Gansu | Sub-pop2 |
| 32 | C72-6-1-2-1 | Gansu | Sub-pop2 |
| 33 | Longjian 111 | Gansu | Sub-pop3 |
| 34 | 0052-1-1-4-1 | Gansu | Sub-pop3 |
| 35 | 988-4-2-4-1 | Gansu | Sub-pop2 |
| 36 | 0052-11-2-3 | Gansu | Sub-pop4 |
| 37 | A23-4-3-4-1 | Gansu | Sub-pop4 |
| 38 | 980-4-1-1-2 | Gansu | Sub-pop4 |
| 39 | 9984-3-2-1 | Gansu | Sub-pop2 |
| 40 | C55-3-1-1 | Gansu | Sub-pop2 |
| 41 | B11-2-3-1-1-2 | Gansu | Sub-pop2 |
| 42 | Longmai 079 | Gansu | Sub-pop2 |
| 43 | E69-4-1 | Gansu | Sub-pop2 |
| 44 | A8-4-4-2 | Gansu | Sub-pop4 |
| 45 | C28-5-1-3 | Gansu | Sub-pop3 |
| 46 | C72-1-3-2-2 | Gansu | Sub-pop2 |
| 47 | C14-14-1 | Gansu | Sub-pop2 |
| 48 | E72-2-2 | Gansu | Sub-pop3 |
| 49 | Hangxuan 01 | Gansu | Sub-pop4 |
| 50 | 1-4-8-1 | Gansu | Sub-pop3 |
| 51 | Hangxuan 121 | Gansu | Sub-pop1 |
| 52 | 0052-13 | Gansu | Sub-pop3 |
| 53 | B61-2-3-2-1 | Gansu | Sub-pop3 |
| 54 | A80-3-1-1-1 | Gansu | Sub-pop2 |
| 55 | B17-2-3-2-1 | Gansu | Sub-pop3 |
| 56 | Chang 721 | Shanxi | Sub-pop2 |
| 57 | Longmai 838 | Gansu | Sub-pop2 |
| 58 | Chang 844 | Shanxi | Sub-pop2 |
| 59 | B61-2-1-2-2 | Gansu | Sub-pop4 |
| 60 | Chang 9325 | Shanxi | Sub-pop1 |
| 61 | C130-5-1-1 | Gansu | Sub-pop3 |
| 62 | A80-4-1-1-1 | Gansu | Sub-pop2 |
| 63 | Lantian 13 | Gansu | Sub-pop3 |
| 64 | Longmai 844 | Gansu | Sub-pop3 |
| 65 | Dd52-17-3-2 | Gansu | Sub-pop3 |
| 66 | 1-1-2-7 | Gansu | Sub-pop3 |
| 67 | 0052-12-7-3 | Gansu | Sub-pop3 |
| 68 | Lantian 15 | Gansu | Sub-pop4 |
| 69 | 0052-17-2 | Gansu | Sub-pop3 |
| 70 | 27--1 | Gansu | Sub-pop2 |
| 71 | 0052-12-1-2 | Gansu | Sub-pop2 |
| 72 | 00-71 | Gansu | Sub-pop1 |
| 73 | A88-4-2-4 | Gansu | Sub-pop2 |
| 74 | Chang 4758 | Shanxi | Sub-pop3 |
| 75 | 29--2-2 | Gansu | Sub-pop3 |
| 76 | Longmai 847 | Gansu | Sub-pop4 |
| 77 | Longjian 4 | Gansu | Sub-pop1 |
| 78 | Longjian 104 | Gansu | Sub-pop3 |
| 79 | Longyu 5 | Gansu | Sub-pop1 |
| 80 | Longjian 103 | Gansu | Sub-pop2 |
| 81 | Longjian 169 | Gansu | Sub-pop3 |
| 82 | Dong 03-07 | Gansu | Sub-pop3 |
| 83 | Xindong 18 | Gansu | Sub-pop3 |
| 84 | Lanxuan 1 | Gansu | Sub-pop4 |
| 85 | Jinmai 47 | Shanxi | Sub-pop1 |
| 86 | Jinmai 79 | Shanxi | Sub-pop1 |
| 87 | Longyuan 031 | Gansu | Sub-pop3 |
| 88 | Linfeng 3 | Shanxi | Sub-pop2 |
| 89 | Longyuan 937 | Gansu | Sub-pop3 |
| 90 | Chang 6878 | Shanxi | Sub-pop4 |
| 91 | Yunhan 23-35 | Shanxi | Sub-pop3 |
| 92 | Jinmai 68 | Shanxi | Sub-pop1 |
| 93 | Lumai 14 | Shandong | Sub-pop3 |
| 94 | Yunhan 22-33 | Shanxi | Sub-pop3 |
| 95 | Jindong 8 | Beijing | Sub-pop1 |
| 96 | Yunhan 2028 | Shanxi | Sub-pop2 |
| 97 | Jing 411 | Beijing | Sub-pop4 |
| 98 | Jinmai 63 | Shanxi | Sub-pop4 |
| 99 | Jintai 170 | Shanxi | Sub-pop1 |
| 100 | Longyuan 961 | Gansu | Sub-pop2 |
| 101 | Linhan 6 | Shanxi | Sub-pop3 |
| 102 | Longyuan 964 | Gansu | Sub-pop3 |
| 103 | Longzhong 2 | Gansu | Sub-pop2 |
| 104 | Yunhan 20410 | Shanxi | Sub-pop1 |
| 105 | Jin 2148-7 | Shanxi | Sub-pop2 |
| 106 | Chang 6452 | Shanxi | Sub-pop3 |
| 107 | Longzimai 1 | Gansu | Sub-pop4 |
| 108 | Jinmai 72 | Shanxi | Sub-pop4 |
| 109 | Pubing 151 | Gansu | Sub-pop4 |
| 110 | Yumai 18 | Henan | Sub-pop1 |
| 111 | 1R8 | America | Sub-pop1 |
| 112 | 1R14 | America | Sub-pop3 |
| 113 | 1R20 | America | Sub-pop4 |
| 114 | 1R19 | America | Sub-pop4 |
| 115 | 0052-1-4-1 | Gansu | Sub-pop2 |
| 116 | 0052-1-6-1 | Gansu | Sub-pop3 |
| 117 | Ningmai 5 | Gansu | Sub-pop2 |
| 118 | 1R6 | America | Sub-pop3 |
| 119 | 1R5 | America | Sub-pop4 |
| 120 | 1R1 | America | Sub-pop4 |
| 121 | Q9086 | Gansu | Sub-pop3 |
| 122 | 9840-0-3-2 | Gansu | Sub-pop2 |
| 123 | Chang 6738 | Shanxi | Sub-pop1 |
| 124 | Longjian 127 | Gansu | Sub-pop2 |
| 125 | Longjian 3 | Gansu | Sub-pop4 |
| 126 | Qingnong 3 | Gansu | Sub-pop4 |
| 127 | 1R39 | America | Sub-pop1 |
| 128 | 1R38 | America | Sub-pop2 |
| 129 | 1R27 | America | Sub-pop4 |
| 130 | 1R25 | America | Sub-pop2 |
| 131 | 1R26 | America | Sub-pop2 |
| 132 | 1R2 | America | Sub-pop3 |
| 133 | 0052-1-3 | Gansu | Sub-pop4 |
| 134 | 1R17 | America | Sub-pop1 |
| 135 | Qingnong 4 | Gansu | Sub-pop3 |
| 136 | Longjian 301 | Gansu | Sub-pop1 |
| 137 | Longjian 386 | Gansu | Sub-pop3 |
| 138 | Changhan 4738 | Shaanxi | Sub-pop4 |
| 139 | 1R11 | America | Sub-pop1 |
| 140 | 94164-1 | Gansu | Sub-pop4 |
| 141 | Longjian 387 | Gansu | Sub-pop1 |
| 142 | Longjian 385 | Gansu | Sub-pop1 |
| 143 | Yunhan 2129 | Shanxi | Sub-pop2 |
| 144 | Linhan 234 | Shanxi | Sub-pop2 |
| 145 | Linhan 538 | Shanxi | Sub-pop2 |
| 146 | Linhan 21241 | Shanxi | Sub-pop2 |
| 147 | Baiqimai | Gansu | Sub-pop1 |
| 148 | Beijing 8686 | Beijing | Sub-pop4 |
| 149 | Cangmai 6001 | Hebei | Sub-pop3 |
| 150 | Changlei 5 | Shandong | Sub-pop3 |
| 151 | Chang 4640 | Shanxi | Sub-pop4 |
| 152 | Chang 4738 | Shanxi | Sub-pop3 |
| 153 | Chang 6154 | Shanxi | Sub-pop2 |
| 154 | Chang 6359 | Shanxi | Sub-pop2 |
| 155 | Qingshan 782 | Gansu | Sub-pop4 |
| 156 | Qingshan 821 | Gansu | Sub-pop4 |
| 157 | Chang 8744 | Shanxi | Sub-pop4 |
| 158 | Han 4589 | Hebei | Sub-pop4 |
| 159 | Hanxuan 10 | Beijing | Sub-pop2 |
| 160 | Heng 7228 | Hebei | Sub-pop2 |
| 161 | Heng 95 Guan 26 | Hebei | Sub-pop2 |
| 162 | Hengyou 18 | Hebei | Sub-pop1 |
| 163 | Jimai 21 | Shandong | Sub-pop2 |
| 164 | Jimai 32 | Hebei | Sub-pop4 |
| 165 | Qingshan 843 | Gansu | Sub-pop3 |
| 166 | Qingshan 851 | Gansu | Sub-pop3 |
| 167 | Tao 157 | Gansu | Sub-pop3 |
| 168 | Jinnong 207 | Shanxi | Sub-pop3 |
| 169 | Xifeng 27 | Gansu | Sub-pop3 |
| 170 | Xifeng 28 | Gansu | Sub-pop2 |
| 171 | Xiping 1 | Gansu | Sub-pop4 |
| 172 | Jingshuang 2 | Beijing | Sub-pop4 |
| 173 | Keyi 29 | Tianjin | Sub-pop4 |
| 174 | Xiannong 4 | Shaanxi | Sub-pop4 |
| 175 | Linfeng 615 | Shanxi | Sub-pop4 |
| 176 | Longjian 196 | Gansu | Sub-pop2 |
| 177 | Longjian 294 | Gansu | Sub-pop4 |
| 178 | Lude 1 | Shandong | Sub-pop4 |
| 179 | Lumai 1 | Shandong | Sub-pop1 |
| 180 | Chang 4378 | Shanxi | Sub-pop1 |
| 181 | Lumai 15 | Shandong | Sub-pop1 |
| 182 | Lunkang 7 | Tianjin | Sub-pop1 |
| 183 | Lunxuan 987 | Beijing | Sub-pop1 |
| 184 | Shi 4185 | Hebei | Sub-pop1 |
| 185 | Shijiazhuang 8 | Hebei | Sub-pop2 |
| 186 | Shimai 12 | Hebei | Sub-pop2 |
| 187 | Shimai 13 | Hebei | Sub-pop4 |
| 188 | Silenghonghulutou | Hebei | Sub-pop2 |
| 189 | Xifeng 16 | Gansu | Sub-pop2 |
| 190 | Xifeng 20 | Gansu | Sub-pop4 |
| 191 | Xinong 688 | Shaanxi | Sub-pop1 |
| 192 | Xinong 797 | Shaanxi | Sub-pop4 |
| 193 | Xinong 979 | Shaanxi | Sub-pop4 |
| 194 | Yunhan 21-30 | Shanxi | Sub-pop2 |
| 195 | A80-4-2-1-2-1 | Gansu | Sub-pop1 |
| 196 | A8-4-4-2 | Gansu | Sub-pop4 |
| 197 | A80-3-2-2-1 | Gansu | Sub-pop2 |
| 198 | 0052-17-2-2 | Gansu | Sub-pop2 |

**Supplementary Table 2.** Sequences of primers for qRT-PCR.

| **Gene ID** | **Forward** | **Reverse** |
| --- | --- | --- |
| TraesCS5D02G011900 | GAGATTGGTGTGTGCGGATC | GTTGTTGGTATGATCCGCCG |
| TraesCS5D02G223200 | CCTGAGTGATGACCTCCCTC | TGGACCGACAGACACTCAAA |
| TraesCS5D02G223700 | ATCAGTACAGAGGGATCCGC | GCCTTCTTGCCACGAATCTT |
| TraesCS1A02G135300 | ATTCCGCCGTCTTAATGTGC | TTGCAGCCCAATGAATAGCC |
| TraesCS5B02G428200 | ACCTTCAAGATCTCCGCCTC | AGGTCTGTTCGCTGGTTAGT |
| TraesCS6B02G323800LC | TCCGCTGGATGATCACTGG | TGAACCCCTGGCTCGTAGTA |
| TraesCS7D02G535400 | GCAAGTTCTCTGTTCCCGTG | AGAACTCTTCAAGGCCTCCC |
| TaActin | GACCCAGACAACTCGCAAC | GGAATCCATGACCACCTAC |

**Supplementary Table 3.** Summary of markers significantly associated with the kernel traits.

| **Trait^a^** | **SNP Marker** | **Allele** | **Chr^b^** | **Postion(bp)** | **Environment** | ***P*-Value** | ***R*^2c^(%)** |
| --- | --- | --- | --- | --- | --- | --- | --- |
| KL | AX-95225890 | G/T | 1D | 382635710 | E3 | 5.39E-04 | 7.94 |
| KL | AX-94814715 | G/T | 2D | 114511017 | E6 | 9.62E-04 | 7.35 |
| KL | AX-94887053 | G/C | 3A | 13555700 | BLUP | 7.38E-04 | 7.99 |
| KL | AX-94710887 | T/G | 3D | 613735402 | E6 | 1.21E-04 | 10.42 |
| KL | AX-95108463 | C/G | 4A | 602261189 | E6 | 3.31E-04 | 8.84 |
| KL | AX-94649016 | A/C | 4D | 337074037 | E2 | 4.84E-04 | 8.90 |
| KL | AX-94744774 | T/C | 4D | 488650307 | E6 | 5.08E-04 | 8.05 |
| KL | AX-94477926 | C/A | 5B | 384720486 | BLUP | 8.69E-04 | 7.73 |
| KL | AX-95248961 | A/T | 5D | 6450092 | E5 | 6.41E-04 | 7.91 |
| KL | AX-95248961 | A/T | 5D | 6450092 | E6 | 2.40E-04 | 8.91 |
| KL | AX-95248961 | A/T | 5D | 6450092 | BLUP | 6.88E-04 | 7.80 |
| KL | AX-94400331 | C/T | 5D | 330891375 | E4 | 3.77E-04 | 8.76 |
| KL | AX-94400331 | C/T | 5D | 330891375 | BLUP | 4.81E-04 | 8.44 |
| KL | AX-94509671 | A/C | 5D | 331402475 | E2 | 2.25E-04 | 8.99 |
| KL | AX-94509671 | A/C | 5D | 331402475 | E3 | 6.67E-04 | 7.69 |
| KL | AX-94509671 | A/C | 5D | 331402475 | E4 | 4.57E-04 | 8.20 |
| KL | AX-94509671 | A/C | 5D | 331402475 | E5 | 7.87E-04 | 7.57 |
| KL | AX-94509671 | A/C | 5D | 331402475 | BLUP | 3.08E-05 | 11.23 |
| KL | AX-94905663 | T/C | 6A | 69138326 | E6 | 7.74E-04 | 6.34 |
| KL | AX-94969710 | A/G | 6A | 555580233 | E5 | 8.30E-04 | 7.77 |
| KL | AX-94735973 | T/C | 6B | 465878200 | E5 | 4.40E-04 | 8.41 |
| KL | AX-94450273 | C/T | 7D | 29834228 | E1 | 2.91E-04 | 6.83 |
| KW | AX-94393836 | C/T | 1A | 208219190 | E1 | 7.78E-06 | 14.68 |
| KW | AX-94393836 | C/T | 1A | 208219190 | E5 | 1.13E-04 | 10.62 |
| KW | AX-94393836 | C/T | 1A | 208219190 | BLUP | 4.59E-06 | 15.02 |
| KW | AX-95185443 | T/C | 1B | 587984409 | E6 | 3.02E-04 | 8.99 |
| KW | AX-94402876 | G/A | 2A | 757885904 | E1 | 4.42E-04 | 8.91 |
| KW | AX-94658428 | G/A | 2A | 79202527 | E1 | 1.82E-04 | 7.83 |
| KW | AX-94663384 | A/G | 2A | 773218951 | E5 | 3.62E-04 | 8.55 |
| KW | AX-94682149 | T/C | 2A | 640225089 | BLUP | 5.48E-04 | 8.09 |
| KW | AX-94820072 | A/C | 2A | 197542607 | E3 | 6.79E-04 | 10.13 |
| KW | AX-94484022 | C/G | 2B | 176705865 | E2 | 3.48E-04 | 6.95 |
| KW | AX-94622328 | T/G | 2B | 162928030 | E6 | 4.37E-04 | 8.04 |
| KW | AX-94958164 | A/G | 2B | 34419207 | E5 | 1.72E-04 | 10.07 |
| KW | AX-94995929 | C/T | 2B | 184331481 | E2 | 9.43E-04 | 7.47 |
| KW | AX-94577392 | G/A | 2D | 513021548 | BLUP | 2.81E-04 | 9.26 |
| KW | AX-95170089 | A/G | 2D | 617128314 | E2 | 5.60E-04 | 8.53 |
| KW | AX-94732009 | A/T | 2D | 629402855 | E5 | 1.93E-04 | 7.55 |
| KW | AX-94710887 | T/G | 3D | 613735402 | E6 | 4.29E-04 | 8.98 |
| KW | AX-94731719 | C/A | 3D | 603975980 | E2 | 7.97E-04 | 8.19 |
| KW | AX-94554181 | A/G | 5A | 571789461 | E5 | 4.55E-05 | 9.13 |
| KW | AX-94664659 | C/T | 5B | 13361005 | BLUP | 7.81E-04 | 9.19 |
| KW | AX-95188907 | A/G | 5B | 304764997 | E1 | 5.97E-04 | 9.04 |
| KW | AX-94477071 | C/G | 5B | 515193552 | E6 | 9.85E-04 | 7.63 |
| KW | AX-94488049 | C/T | 5B | 707707827 | E6 | 6.47E-04 | 7.83 |
| KW | AX-94438072 | C/T | 5D | 561626204 | E3 | 7.14E-04 | 6.14 |
| KW | AX-94438072 | C/T | 5D | 561626204 | E5 | 6.86E-04 | 6.36 |
| KW | AX-94438072 | C/T | 5D | 561626204 | BLUP | 2.63E-04 | 7.35 |
| KW | AX-94968296 | T/C | 6A | 23411883 | E6 | 4.60E-04 | 8.43 |
| KW | AX-94963438 | G/A | 6A | 611563774 | E6 | 5.49E-04 | 7.99 |
| KW | AX-94433019 | C/G | 6A | 613976303 | E6 | 1.75E-04 | 7.99 |
| KW | AX-94592155 | C/T | 6B | 151378744 | E4 | 3.97E-04 | 9.05 |
| KW | AX-94647071 | G/T | 6B | 155237535 | E4 | 4.23E-04 | 7.18 |
| KW | AX-95115165 | T/C | 6D | 11059665 | E2 | 8.71E-04 | 7.78 |
| KW | AX-95111184 | T/C | 6D | 239807586 | E2 | 5.55E-04 | 6.31 |
| KW | AX-94412202 | C/T | 7A | 730749845 | E4 | 9.29E-04 | 6.34 |
| KW | AX-94440608 | C/T | 7A | 562001673 | E5 | 5.89E-04 | 8.27 |
| KW | AX-94673769 | T/C | 7A | 624807841 | E4 | 5.87E-04 | 9.69 |
| KW | AX-94389673 | A/G | 7A | 699740214 | E6 | 3.49E-04 | 6.77 |
| KW | AX-94535036 | G/A | 7A | 732921487 | E4 | 4.06E-04 | 8.93 |
| KW | AX-95023072 | G/T | 7B | 3364326 | BLUP | 8.68E-04 | 7.63 |
| KW | AX-94426964 | A/G | 7B | 27346800 | E2 | 4.21E-04 | 8.37 |
| KW | AX-94918120 | G/T | 7B | 557060624 | BLUP | 8.80E-04 | 8.02 |
| KW | AX-94471749 | G/A | 7B | 652928863 | BLUP | 7.31E-04 | 8.82 |
| KW | AX-95247039 | G/A | 7D | 616509365 | E4 | 4.41E-04 | 6.79 |
| KDR | AX-94843910 | T/C | 1A | 29191532 | E5 | 9.92E-04 | 7.89 |
| KDR | AX-94729286 | A/G | 1D | 491051266 | E1 | 6.55E-04 | 6.02 |
| KDR | AX-94655931 | C/T | 2A | 459506834 | E2 | 3.34E-04 | 6.93 |
| KDR | AX-94588733 | C/T | 2A | 762318206 | E1 | 5.56E-04 | 8.42 |
| KDR | AX-95260757 | A/G | 2B | 749418335 | E4 | 1.68E-04 | 9.57 |
| KDR | AX-94796017 | T/C | 3A | 22616289 | E1 | 4.50E-04 | 6.38 |
| KDR | AX-94409249 | A/G | 3B | 126000659 | E1 | 5.21E-04 | 8.13 |
| KDR | AX-94409249 | A/G | 3B | 126000659 | E6 | 8.73E-04 | 7.81 |
| KDR | AX-94409249 | A/G | 3B | 126000659 | BLUP | 6.46E-04 | 8.19 |
| KDR | AX-94687911 | T/G | 3B | 145900438 | E2 | 5.83E-05 | 9.13 |
| KDR | AX-94670667 | T/G | 3B | 479876161 | E2 | 5.19E-04 | 8.05 |
| KDR | AX-94686079 | T/G | 3D | 84485896 | E6 | 1.93E-04 | 9.46 |
| KDR | AX-95002863 | C/T | 4A | 180406074 | E2 | 7.30E-04 | 7.65 |
| KDR | AX-95139001 | C/G | 4D | 403201345 | E1 | 6.74E-04 | 7.06 |
| KDR | AX-94730953 | G/T | 5A | 226209499 | E5 | 5.15E-04 | 8.99 |
| KDR | AX-94927526 | G/T | 5A | 599218786 | E2 | 2.40E-04 | 8.83 |
| KDR | AX-94826800 | T/G | 5B | 35976710 | E2 | 8.01E-05 | 10.73 |
| KDR | AX-94421372 | C/T | 5B | 550229479 | E4 | 5.33E-04 | 8.42 |
| KDR | AX-95176386 | A/G | 5B | 601325605 | E1 | 6.78E-04 | 8.58 |
| KDR | AX-95629937 | G/A | 5B | 604022658 | E4 | 5.44E-05 | 11.90 |
| KDR | AX-95629937 | G/A | 5B | 604022658 | BLUP | 1.72E-04 | 10.38 |
| KDR | AX-95248961 | A/T | 5D | 6450092 | E6 | 4.50E-05 | 10.80 |
| KDR | AX-94509671 | A/C | 5D | 331402475 | E2 | 5.42E-04 | 7.93 |
| KDR | AX-94950506 | C/T | 6A | 6733332 | E2 | 9.91E-04 | 7.93 |
| KDR | AX-94905663 | T/C | 6A | 69138326 | E6 | 4.36E-04 | 6.88 |
| KDR | AX-94428441 | T/C | 6B | 20999197 | E2 | 5.82E-04 | 9.10 |
| KDR | AX-94711022 | A/G | 6B | 252383444 | E3 | 6.42E-04 | 6.32 |
| KDR | AX-94711022 | A/G | 6B | 252383444 | E4 | 7.36E-04 | 6.06 |
| KDR | AX-95243020 | T/C | 6B | 267022749 | E3 | 7.24E-04 | 6.07 |
| KDR | AX-94735973 | T/C | 6B | 465878200 | E6 | 6.99E-04 | 7.93 |
| KDR | AX-94756372 | A/G | 6B | 475272891 | E3 | 8.25E-04 | 7.66 |
| KDR | AX-94646382 | A/G | 7B | 353967624 | E3 | 7.46E-04 | 6.57 |
| KDR | AX-94632774 | T/C | 7B | 518460302 | E3 | 2.33E-04 | 9.30 |
| KDR | AX-94907048 | G/A | 7B | 586631421 | E4 | 1.55E-04 | 9.49 |
| KDR | AX-94578940 | A/C | 7D | 627323708 | E3 | 4.63E-04 | 6.58 |
| KDR | AX-94578940 | A/C | 7D | 627323708 | BLUP | 8.40E-04 | 5.91 |
| KP | AX-94467726 | A/G | 1A | 544055323 | E4 | 8.65E-04 | 7.77 |
| KP | AX-94622328 | T/G | 2B | 162928030 | E6 | 5.63E-04 | 7.94 |
| KP | AX-94430710 | A/G | 2B | 172984373 | E6 | 8.18E-04 | 7.53 |
| KP | AX-94895053 | T/C | 2D | 30335972 | E6 | 8.21E-04 | 7.95 |
| KP | AX-94571864 | C/T | 2D | 69251469 | E2 | 9.52E-04 | 7.54 |
| KP | AX-94667578 | A/G | 2D | 69504702 | E4 | 6.28E-04 | 8.22 |
| KP | AX-94814715 | G/T | 2D | 114511017 | E6 | 5.91E-04 | 7.90 |
| KP | AX-94887053 | G/C | 3A | 13555700 | BLUP | 4.66E-04 | 8.51 |
| KP | AX-94911783 | C/T | 3A | 648868027 | E1 | 3.68E-04 | 8.99 |
| KP | AX-94710887 | T/G | 3D | 613735402 | E6 | 4.58E-05 | 11.73 |
| KP | AX-95108463 | C/G | 4A | 602261189 | E6 | 2.72E-04 | 9.12 |
| KP | AX-94425015 | C/G | 4B | 2036666 | E4 | 2.55E-04 | 8.83 |
| KP | AX-94632604 | A/C | 4B | 2036703 | E4 | 1.83E-04 | 9.22 |
| KP | AX-94576171 | G/A | 4B | 636779367 | BLUP | 8.97E-04 | 7.75 |
| KP | AX-94744774 | T/C | 4D | 488650307 | E6 | 4.10E-04 | 8.29 |
| KP | AX-94414339 | G/A | 5A | 556006342 | E2 | 8.28E-04 | 7.81 |
| KP | AX-95221652 | A/G | 5A | 560462508 | E4 | 4.29E-04 | 6.70 |
| KP | AX-94791648 | C/T | 5B | 356184533 | E1 | 3.54E-04 | 8.97 |
| KP | AX-94400331 | C/T | 5D | 330891375 | E4 | 9.09E-04 | 7.75 |
| KP | AX-94400331 | C/T | 5D | 330891375 | BLUP | 7.48E-04 | 7.89 |
| KP | AX-94509671 | A/C | 5D | 331402475 | E2 | 5.55E-04 | 7.97 |
| KP | AX-94509671 | A/C | 5D | 331402475 | BLUP | 5.72E-05 | 10.49 |
| KP | AX-94450273 | C/T | 7D | 29834228 | E1 | 7.16E-05 | 8.45 |
| TKW | AX-94393836 | C/T | 1A | 208219190 | E4 | 1.73E-04 | 10.66 |
| TKW | AX-94393836 | C/T | 1A | 208219190 | E5 | 5.95E-04 | 8.68 |
| TKW | AX-94945293 | T/C | 1A | 582294879 | E4 | 2.36E-04 | 9.01 |
| TKW | AX-94862571 | G/A | 1B | 673958493 | BLUP | 2.32E-04 | 9.24 |
| TKW | AX-95633318 | A/G | 2A | 97308160 | E4 | 8.29E-04 | 8.65 |
| TKW | AX-94560128 | C/G | 2A | 780714708 | E4 | 7.13E-04 | 7.71 |
| TKW | AX-94958164 | A/G | 2B | 34419207 | E5 | 4.85E-04 | 9.20 |
| TKW | AX-94484022 | C/G | 2B | 176705865 | E2 | 9.44E-04 | 5.95 |
| TKW | AX-94526347 | C/T | 2B | 245130174 | BLUP | 9.69E-04 | 7.54 |
| TKW | AX-95632731 | G/A | 3A | 659398759 | BLUP | 3.26E-04 | 9.14 |
| TKW | AX-94631203 | T/G | 5A | 17222661 | BLUP | 5.27E-04 | 8.27 |
| TKW | AX-94554181 | A/G | 5A | 571789461 | E5 | 7.42E-04 | 6.10 |
| TKW | AX-95015210 | T/A | 5B | 15186271 | BLUP | 5.53E-04 | 8.07 |
| TKW | AX-95072670 | A/C | 5B | 15197261 | BLUP | 5.59E-04 | 8.00 |
| TKW | AX-94560600 | T/C | 5B | 17574984 | BLUP | 8.62E-04 | 7.64 |
| TKW | AX-94559061 | G/C | 5B | 17968963 | BLUP | 4.71E-04 | 8.33 |
| TKW | AX-94414150 | C/T | 5B | 17969070 | BLUP | 6.69E-04 | 7.89 |
| TKW | AX-94819081 | C/T | 5D | 173610156 | E3 | 5.87E-04 | 8.74 |
| TKW | AX-95247754 | C/T | 6B | 270871889 | E1 | 6.55E-04 | 6.39 |
| TKW | AX-95192704 | G/A | 6B | 470127283 | E4 | 4.42E-04 | 8.47 |
| TKW | AX-94384008 | C/T | 7A | 3425130 | BLUP | 4.68E-04 | 8.92 |
| TKW | AX-94440608 | C/T | 7A | 562001673 | E5 | 2.70E-04 | 9.17 |

^a^ KL: kernel length; KW: kernel width; KDR: kernel diameter ratio; KP: kernel perimeter ; TKW: thousand kernel weight. ^b^ Chr: Chromosome. ^c^ Phenotypic variance explained by the MTAs.
